# Navigating Social Waters: Understanding Theory-of-Mind Challenges in Patients with Mesial Temporal Lobe Epilepsy

**DOI:** 10.3390/jcm13051410

**Published:** 2024-02-29

**Authors:** Aleksandra Bala, Agnieszka Olejnik, Maria Mojżeszek, Andrzej Rysz, Przemysław Kunert

**Affiliations:** 1 Faculty of Psychology, University of Warsaw, Stawki 5/7, 00-183 Warsaw, Poland; 2 Department of Neurosurgery, Medical University of Warsaw, Banacha 1a, 02-097 Warsaw, Poland

**Keywords:** epilepsy, MTLE, social cognition, theory of mind, neuropsychology

## Abstract

**Background:** Temporal lobe epilepsy is a common neurological disease that affects many areas of patients’ lives, including social competence. The aim of the study was to assess theory of mind in patients with temporal lobe epilepsy and to investigate the demographic and clinical factors associated with this function. **Methods:** A total of 65 participants took part in the study, which included 44 patients with epilepsy and 21 demographically matched healthy individuals. The following neuropsychological tests were used to examine theory of mind: the Faux Pas Test, the Hinting Task, the Emotion Comprehension Test, and a cognitive function screen, the Montreal Cognitive Assessment. **Results:** Patients with epilepsy scored lower on all measures of the theory-of-mind tests. Moreover, in the clinical group, numerous moderate and strong correlations were found between the theory-of-mind tests and education, age at onset of epilepsy, lateralization of epileptic focus, cognitive status, and, to a lesser degree, number of anti-epileptic drugs, frequency of seizures, and age. In contrast, in the control group, significant correlations were found mostly between the theory-of-mind tests and sex, and, to a lesser degree, age. Education and cognitive functioning were not associated. **Conclusions:** Patients with epilepsy experience difficulties in theory of mind, which may have a negative impact on the quality of their social relationships. The level of theory-of-mind abilities correlates with particular clinical and demographic indicators. Recognizing these issues allows clinicians to implement tailored interventions, potentially improving patients’ quality of life.

## 1. Introduction

Epilepsy is a common disorder of the central nervous system (CNS) [1] that affects about 70 million individuals globally [2]. Temporal lobe epilepsy (TLE) is one of the most common types of focal epilepsies. TLE is divided into two subtypes: epilepsy with a focus in the lateral part of the temporal lobe (lateral temporal lobe epilepsy [LTLE]) and epilepsy with a focus in the mesial part of the temporal lobe, mainly in the area of the hippocampus, amygdala, or entorhinal cortex (mesial temporal lobe epilepsy [MTLE]) [3,4]. Among patients with TLE, approximately 50–70% are resistant to pharmacological treatment [5], which means that epileptic seizures do not stop under the influence of medication and continuously affect numerous areas of functioning. The structures affected in MTLE are crucial for effective functioning; thus, difficulties in cognitive, social, and emotional functioning are often observed [6].

One of the issues that has been increasingly discussed in the context of patients with epilepsy is social cognition. It is difficult to find a universal and commonly used definition of social cognition. Most often, this concept is divided into several cognitive subsystems, such as theory of mind (ToM), social perception, social knowledge, attributional bias, and emotional processing [7]. The research indicates that the brain structures involved in social cognition include parts of the temporal lobe, specifically the posterior superior temporal sulcus, temporo-parietal junction, temporal poles, fusiform gyrus, and amygdala, as well as its connections with the prefrontal cortex and the cingulate cortex [8,9]. Patients with MTLE often exhibit deficits in social cognition due to the involvement of temporal structures in the neural networks that are crucial for understanding social cues. Seizures and structural abnormalities in this area may disrupt the intricate connections required for ToM, impairing the ability to interpret social nuances and recognize the emotions and intentions of others. The literature points to the presence of difficulties with processing social information, recognizing facial expressions, detecting gaze direction, social perception, and ToM [10,11,12,13,14].

As mentioned before, one of the main aspects of social cognition is ToM. The term “theory of mind” was first used in the work of Premack and Woodruff, who studied the ability to mentalize in primates [15]. This has given rise to many studies involving people originating from various clinical populations. ToM can be characterized as the capacity to assign cognitive and emotional mental states, such as desires and beliefs, to others. Consequently, processes involving mentalization play a pivotal role in comprehending the behavior of individuals and in crafting appropriate responses within intricate social scenarios [16]. ToM also allows for the collection of relevant information about the surrounding world. It provides clues as to how we understand the behavior of others and how to act ourselves [17]. ToM consists of two components: decoding mental states and resonating with them. Decoding involves perceiving and recognizing certain states of others, e.g., facial expressions, body language, indirect verbal responses, and conversational subtext. The second component of ToM is resonating, which involves predicting other people’s behavior based on their mental state [18]. These components make ToM crucial for proper social functioning, and deficits in this field can make interpersonal contact significantly challenging [19].

A number of studies indicate difficulties in ToM in patients with various CNS dysfunctions. Some researchers emphasize the underperformance of multiple sclerosis patients in verbal and non-verbal tests when examining ToM compared to the healthy population [20]. Similar difficulties are found in patients with Alzheimer’s disease [21] or those with an acquired brain injury [22]. Researchers have also explored ToM in individuals with epilepsy. The social life of patients with epilepsy is challenging. In addition to objective difficulties, they face stigmatization and prejudices that make it challenging to establish social relations and practice social skills [23,24]. The experienced difficulties may also be associated with deficits in social cognition. Previous research has shown that patients with TLE achieve lower results on ToM tests than healthy individuals [25,26,27]. Neuroimaging studies indicate that the structural and functional changes that occur in TLE may explain the neurological background of these impairments [28].

There are still some inconsistent findings on ToM dysfunction in patients with TLE. There is no consensus among researchers on the clinical factors influencing the degree of ToM impairment. For instance, Hennion and associates [29] observed that ToM difficulties in patients with TLE occur independently of factors such as time of epilepsy onset, gender, or location of epileptogenic focus; however, this is not consistent with the findings of Shaw and colleagues [30], who indicate that early onset of seizures, which is associated with damage to the amygdala and other mesial temporal structures, is associated with greater ToM dysfunction. Although more is known regarding ToM, further research is needed to help explain the nature of ToM deficits in patients with epilepsy and to determine risk factors for the development of these difficulties.

In our study, we focused on the comprehensive evaluation of ToM skills in patients with MTLE. We hypothesized that they would score lower on all aspects of ToM compared to the healthy population. We also expected that the ToM scores would correlate with the demographic and clinical indicators in the clinical group. 

## 2. Materials and Methods

### 2.1. Participants

Sixty-five participants took part in this cross-sectional study. The group consisted of 44 patients with MTLE and 21 healthy volunteers. Subjects from the clinical group were invited to participate in the study during a standard-diagnostics hospitalization. The inclusion criterion for the clinical group was a diagnosis of MTLE confirmed according to neurological examination. The diagnosis of MTLE was based on the phenomenology of the seizures, confirmed with the use of video EEG, MRI, and positron emission tomography. Demographically matched healthy participants were invited to participate in the study through announcements on social media. The exclusion criteria for both groups were a history of any neurological (other than epilepsy in the clinical group) or psychiatric treatment, drug or alcohol addiction, severe cognitive impairment (MoCA test less than 26 points), and physical disabilities that would affect the patient’s ability to complete the task properly (e.g., major visual deficit). The demographic and clinical characteristics of the participants are presented in Table 1.

### 2.2. Diagnostic Tools

**The Faux Pas Test** [31] is a psychological tool used for the evaluation of ToM, specifically the ability to understand social nuances and to recognize when a person has made an unintentional social failure (faux pas). In this test, participants are presented with a set of stories that involve social interactions in which a character unintentionally makes a social error. The participant’s task is to identify and explain the faux pas made by the characters. Performance on this test provides insight into one’s ability to recognize and understand social cues, understand intentions, and differentiate between appropriate and inappropriate social behavior. The test contains 10 stories with faux pas and 10 without faux pas. After reading each story, the respondents must answer several questions that allow for the identification of the following indicators of ToM deficits: (a) faux pas detection (Q1 and Q2), (b) understanding faux pas (Q3), (c) intentions (Q4), (d) beliefs (Q5), (e) empathy (Q6), and (f) comprehension (Q7 and Q8). 

**The Hinting Task** [32] is an assessment tool used to evaluate an individual’s ability to understand indirect, subtle social cues or hints. The researcher reads a short story about an interaction between two characters. At the end of each scenario, one character drops a hint or indirectly implies something, and the participants need to interpret the underlying message conveyed by that hint and explain what the character really meant. If the respondent immediately gives the correct answer, he or she receives two points, but if not, he or she receives an additional hint in the form of another sentence explaining the speaker’s intentions and has a second chance to answer; if the subject gives the correct answer, they are scored one point, and if not, they are scored no points.

**The Emotion Comprehension Test** (Test Rozumienia Emocji—TRE, [33]) consists of five subtests that contain various tasks that examine one’s knowledge about emotions and ToM. Each part contains six tasks. In this study, we used two subtests: the fourth and the fifth. In the fourth subtest (TRE4), a situation (e.g., surprise) is presented, for which one should select the feeling/emotional state that has the greatest probability of appearing in the given situation. In the fifth part (TRE5), a situation and the emotional reaction that occurred in it are briefly described. The subject’s task is to choose an answer that describes the circumstances conducive to the occurrence of the reaction presented in the story. In each part, the subject can gain up to six points. 

**The Montreal Cognitive Assessment—MoCA** [34] is a widely used diagnostic tool designed to screen for cognitive impairment. It evaluates various cognitive domains, including attention, concentration, memory, language, visuospatial skills, executive functions, and orientation. The test takes approximately 10–15 min to administer and consists of tasks such as recall, naming, attention, calculation exercises, and clock drawing. The cut-off point for cognitive deficits is a score ≤ 26 of 30 possible points.

### 2.3. Procedure

**Figure 1:**
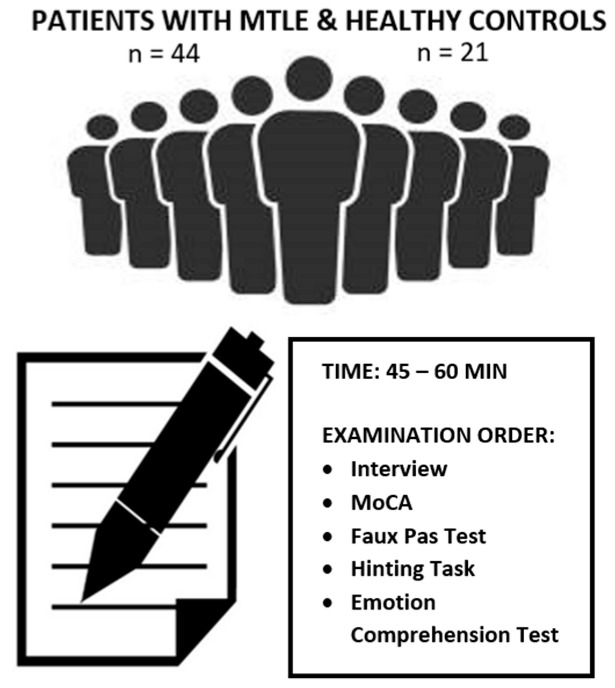
Diagram showing a research overview.

Each participant underwent an individual examination during a single session. The session durations varied (45–60 min) based on the participants’ psychophysical speed. The assessment was conducted in a quiet hospital room. At the beginning of the meeting, participants provided demographic information about themselves. Clinical data, including epilepsy type, course of the disease, and treatment details, were gathered from the patients’ medical records. The Hinting Task, Faux Pas Test, and Emotion Comprehension Test were used to evaluate the patients’ efficacy in terms of ToM (Figure 1). The MoCA test was used to assess general cognitive functioning and to exclude individuals with severe cognitive impairment, which could affect the results of the other diagnostic methods. All the procedures were conducted in accordance with the Declaration of Helsinki and were approved by the ethics committee of the Faculty of Psychology at the University of Warsaw. All participants gave written informed consent to participate in the study. 

### 2.4. Statistical Analysis

The statistical analyses were performed using SPSS version 29.0 (IBM Corp., Armonk, NY, USA) for Windows. The Shapiro–Wilk test showed that the data followed a normal distribution and, as the other requirements were also met, that it was possible to use the parametric tests. The comparisons between the results of the patients with epilepsy and the healthy participants were made using Student’s *t*-test for independent samples. Pearson’s correlation coefficient was used to assess the correlation between the quantitative variables. The η coefficient was used to assess the correlation between nominal and quantitative variables. Results were considered significant when *p* < 0.05.

## 3. Results

First, the scores of the ToM tests obtained from the patients of both groups were compared. The details are shown in Table 2.

The results showed that the groups differed significantly in terms of the points obtained in almost all indicators of the performed tests. The only indicator in which such differences were not observed was the understanding of the neutral aspects of the story in the Faux Pas Test. It is also worth noting that the obtained results had a high and very high effect strength (expressed using Cohen’s d), which exceeded the threshold of 0.8 in all significant results and was over 1 or 2 for some indicators. In the next step, we evaluated whether the obtained results were associated with the clinical and/or demographic variables. For this purpose, correlation analyses were performed separately for the clinical and control groups. The details can be found in Table 3 and Table 4.

The results in the individual Faux Pas subtests were positively correlated with the duration of formal education, age at onset of epilepsy, duration of epilepsy, the number of medications taken, lateralization of the epileptic focus, and the results obtained in the MoCA. Here, moderate and strong correlations prevailed. The scores on the Emotion Comprehension Test correlated with duration of epilepsy, age, and the MoCA test results. The Hinting Task correlated with education, duration of epilepsy, number of seizures per month, and lateralization of the epileptic focus. In the clinical group, no significant correlations were found between the obtained results and gender. As a significant correlation was obtained between the Hinting Task and Faux Pas results and the lateralization of the epileptic focus, an additional analysis was performed to compare the results obtained in these tests for patients with an epileptic focus in the right with those with an epileptic focus in the left hemisphere. The patients with a focus in the left hemisphere achieved lower results in the following indicators: Faux Pas Understanding (t = 1.22; *p* < 0.05), Faux Pas Comprehension (t = 1.84; *p* < 0.05), and the Hinting Task (t = 1.37; *p* < 0.05).

**Figure 2:**
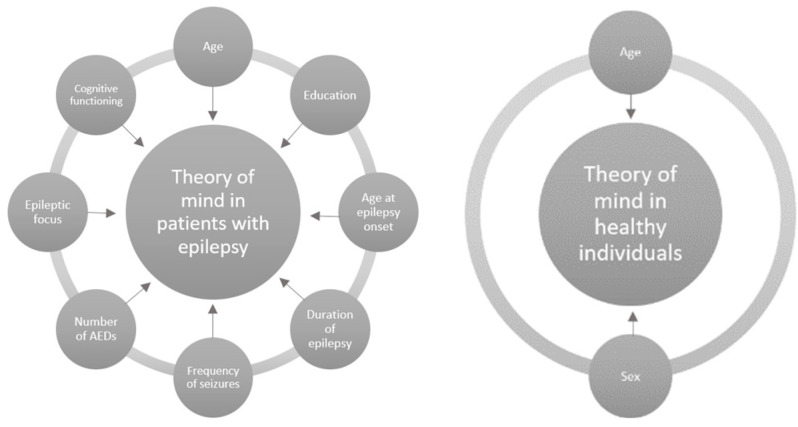
Diagram showing the variables related to the level of ToM in the studied populations.

In the group of healthy participants, significant correlations were observed between the results of selected test indicators comparing ToM with age and gender. No significant correlations were found between the ToM tests and the time of formal education or MoCA scores. Therefore, it has been shown that different variables are associated with the level of ToM performance in patients with epilepsy, as well as in healthy people (Figure 2).

Due to the detection of numerous correlations with gender, an additional analysis was performed to compare the results obtained by women and men. In all analyzed indicators, i.e., Faux Pas Understanding (t = 2.01; *p* < 0.05), Faux Pas Empathy (t = 2.49; *p* < 0.01), Emotion Comprehension Test Situation (t = 1.54; *p* < 0.05), and Emotion Comprehension Test Reaction (t = 1.99; *p* < 0.05), women achieved significantly higher results than men. 

## 4. Discussion

In the present study, we evaluated three components of verbal ToM: the ability to detect the true intentions of a person using subtext, the ability to recognize awkward and socially inappropriate behavior, and the ability to recognize a situation in which someone might behave in a certain way and feel certain emotions. We compared the results of the following two populations: patients with MTLE and healthy volunteers. To increase the precision of the psychometric measurement, we decided to use three different measures: the Faux Pas Test, the Hinting Task, and the Emotion Comprehension Test. A Faux Pas Test involves the patient recognizing violations of certain social norms [35], the Hinting Task focuses on the understanding of another person’s mental state on the basis of indirect speech [36], and the Emotion Comprehension Test measures the empathetic deduction of the patient [33].

Our study showed that patients with MTLE presented impairments in all three evaluated aspects of ToM, which is in line with other studies [26,27,37,38]. Social cognition deficits can be associated with a modified pattern of signal activation in the social cognition network [39]. After a more detailed analysis, we found that the differences did not concern all indicators of the Faux Pas Test but only those regarding ToM. The MTLE patients had problems with detecting a faux pas but also with properly explaining what the gaffe was, what the intentions of the person committing it were, what the knowledge of the person speaking was, and what the person to whom something inappropriate was said felt. There were no differences in understanding the general meaning of the story and their knowledge of neutral facts.

The relationship between select clinical and demographic variables and the results obtained in the ToM tests were analyzed separately in each group. Distinct variables were associated with the results of the ToM tests in the group with epilepsy compared to the healthy participants. In the clinical group, the age at onset of epilepsy, the duration of epilepsy, the lateralization of the epileptic focus, and, to a lesser extent, the frequency of seizures and the number of antiepileptic drugs were significant. In younger patients that started to have seizures, we found that the longer the disease lasted, the lower their scores were on select indicators of the ToM tests, which is in line with some [27,29] but not all studies [26,37,40]. Early onset of the disease may disturb the functioning of the developing brain and prevent the proper growth of various functions [41], including ToM abilities. 

The lower results obtained by people with a left-sided epileptic focus may be related to the modality in which the tasks were presented. All three tests that were used were in text form, where, in addition to ToM, language skills are also important. Numerous studies indicate that people with a left-sided epileptic focus have lower language abilities [42], which could be a secondary impact resulting in the lower results of the ToM tests used in this research. These findings are important, as most of the previous studies show that the right hemisphere is more important for social cognition and that right TLE patients present more severe deficits than those with left TLE [39,43] or that there is no significant difference due to seizure lateralization in social cognition [29,44,45,46]. 

Although some researchers claim that ToM performance is unrelated to the frequency of epileptic seizures or the number of anti-epileptic drugs (AEDs) used [17,37], our study showed that these variables may prove important for at least some aspects of ToM. It is known that pharmacotherapy and seizures have a negative impact on the well-being of patients with epilepsy and can affect their cognitive functioning [41,47]; therefore, this may also have a negative impact on some aspects of their ToM skills.

A significant relationship was also found between ToM and education, general cognitive functioning, and age. Previous studies showed that there is a significant association between ToM and education [27] and between ToM and cognitive functions [48], but most studies did not find any significant association with age [27]. In the case of healthy people, correlations were mostly found between ToM and the gender of the subjects. Women achieved higher scores in many of the examined variables, which is consistent with the results of other studies, indicating a higher social cognition competence in women [49,50]. Interestingly, this effect did not occur in the population of patients with MTLE, which is also in line with other studies [27]. One explanation may be that these current deficits are so large that they balance out the advantage of women, who, under the condition of illness, do not have the opportunity to develop social competence, as is the case in the healthy population. This issue certainly requires further research.

One significant correlation was also noticed in relation to age; older people more accurately indicated the circumstances in which a given reaction could most likely occur, which was consistent with other studies [50]. This was also present in the clinical group. We hypothesize that this is possibly due to accumulated life experiences and exposure to diverse social contexts, regardless of the presence of deficits in this area. Although some studies found no relationship between ToM and age in the clinical population [27] nor in healthy individuals [35,51,52], it is worth emphasizing that none of them used the same diagnostic tool that was used in our study. Contrary to the results obtained from patients with epilepsy, variables such as education or general cognitive functioning were not associated with the ToM performance in the healthy subjects. This may suggest that these abilities are only dependent on intellectual performance to some extent and that the results can improve only up to a certain threshold. Above it, further improvement in intellectual performance is no longer relevant. Similar results can be found in other studies [50].

While the study provides valuable insights into ToM impairment in patients with MTLE, it is essential to acknowledge its limitations. First, the participants in the clinical group were recruited during a standard-diagnostics hospitalization, potentially introducing a bias toward individuals with more severe epilepsy or those seeking medical attention. In further research, it would also be beneficial to include people undergoing outpatient treatment only. The authors of the study wanted to eliminate any factors that could disturb the obtained results; thus, they decided to exclude patients with a significant decline in cognitive functioning from the study. Although this is reasonable and often practiced in neuropsychological research, it may have limited the inclusion of participants with broader cognitive variations. Further research including people with various degrees of cognitive impairment is needed to understand the specificity of the part of the population that was not included in this study. Furthermore, the potential impact of anti-epileptic medications on cognitive functions, including ToM, was not explicitly addressed. Admittedly, the study analyzed the impact of the number of anti-epileptic medications taken by patients on their ToM skills, but due to the size of the group and the huge heterogeneity of the medications, it was not possible to conduct analyses examining the impact of individual substances. Future research could benefit from controlling or analyzing the effects of specific medications on this cognitive domain. The study also excluded people with a history of neurological and psychiatric diseases, but other conditions present in the patients (e.g., hypertension or diabetes) were not taken into account. In the future, if a larger research group is gathered, it would be worth attempting additional analyses that would also take into account the possible relationship of other conditions with the obtained results. Finally, the study participants were from a specific cultural context and may not represent the diversity of cognitive and social experiences in other cultural or ethnic groups. 

Despite significant developments in the available therapeutic options [53], there is still a need for new therapies for epilepsy patients, including neuropsychological therapy. Patients with MTLE face challenges in social competence, which influences various aspects of their lives. Our study identifies significant associations between ToM and clinical/demographic variables such as age at epilepsy onset, localization of epileptic focus, education, cognitive functioning, age, frequency of seizures, and number of AEDs. The obtained results are of great clinical importance, as understanding the specific aspects of ToM deficits in patients with MTLE allows for the development of targeted intervention strategies that focus on enhancing social cognition skills, particularly in areas where patients exhibit the most significant impairments. This tailored approach can potentially improve social functioning and overall quality of life of MTLE patients. Moreover, the study reveals a correlation between a younger age at onset of epilepsy and lower ToM scores. This suggests the importance of early intervention for young patients, which is as soon as they are diagnosed with MTLE. Implementing social cognition interventions at an early stage may mitigate the impact of epilepsy on the development of ToM skills and prevent long-term social challenges. Furthermore, the study results indicate a potential impact of seizure frequency and the number of anti-epileptic drugs on ToM performance. Clinicians managing epilepsy should be aware of these factors and consider their influence on patients’ functioning, as some studies have previously shown that ToM deficits affect patients’ self-appraisal, coping, and overall quality of life [17]. 

Adjustments to treatment plans or additional psychological support may be warranted for patients experiencing more frequent seizures or those taking multiple medications. The study underscores the importance of assessing the ToM skills in patients with MTLE, which was also raised by other researchers previously [17]. If it became a routine part of epilepsy care, this assessment could provide valuable insights for clinicians and enable the earliest possible introduction of social competence training. This would be tailored to the individual needs of particular patients and would take their education and age, as well as the clinical traits of their epilepsy and its treatment, into account. This is a matter of high importance because of its potential positive impact on the well-being of epilepsy patients.

## Figures and Tables

**Table 1:** Demographic and clinical characteristics of the groups.

	MTLE Group *n* = 44	Healthy Controls *n* = 21	*p*
Sex: male/female	20/24	10/11	>0.05
Handedness: R/L	41/3	20/1	>0.05
Age M(SD)	35.70 (10.61)	33.23 (11.49)	>0.05
Years of education M(SD)	13.48 (2.92)	14.00 (1.51)	>0.05
Epilepsy onset M(SD)	13.14 (11.43)	N/A	
Years of epilepsy M(SD)	21.96 (11.06)	N/A	
Numer of seizures/month M(SD)	8.43 (10.63)	N/A	

Legend: M—mean; SD—standard deviation; R—right; L—left; N/A—not applicable.

**Table 2:** Between-group differences in ToM tests.

		Clinical Group *n* = 44	Control Group *n* = 21	Statistics	*p* Value	Cohen’s d
Faux Pas Test	Detection	13.72 (5.83)	34.80 (3.54)	t = 17.99	***p* < 0.001**	4.04
	Understanding	10.52 (3.43)	15.23 (2.38)	t = 6.42	***p* < 0.001**	1.50
	Intentions	10.29 (3.10)	15.04 (2.35)	t = 6.20	***p* < 0.001**	1.64
	Bieliefs	9.70 (3.06)	15.71 (2.43)	t = 7.86	***p* < 0.001**	2.08
	Empathy	10.31 (2.73)	15.85 (2.78)	t = 7.59	***p* < 0.001**	2.01
	Comprehension	33.18 (4.47)	35.00 (3.97)	t = 1.58	*p* = 0.059	0.42
Emotion Comprehension Test	Situation	2.22 (1.64)	4.38 (1.39)	t = 5.18	***p* < 0.001**	1.37
	Reaction	2.25 (1.55)	3.66 (1.71)	t = 3.32	***p* < 0.001**	0.88
Hinting Task		14.45 (4.23)	18.47 (1.56)	t = 5.54	***p* < 0.001**	1.11

Legend: significant results are in bold.

**Table 3:** Correlations between ToM test scores and the clinical and demographic variables in the clinical group.

		Age	Sex	Years of Education	Age at Epilepsy Onset	Seizures/Month	Number of AEDs	Epi Focus	MoCA Scores
Faux Pas Test	Detection	*p* > 0.05	*p* > 0.05	**r = 0.75** ***p* < 0.01**	**r = 0.65** ***p* < 0.05**	*p* > 0.05	**r = −0.19** ***p* < 0.05**	*p* > 0.05	**r = 0.28** ***p* < 0.05**
	Understanding	*p* > 0.05	*p* > 0.05	**r = 0.53** ***p* < 0.05**	**r = 0.48** ***p* < 0.05**	*p* > 0.05	*p* > 0.05	**η = 0.34** ***p* < 0.05**	*p* > 0.05
	Intentions	*p* > 0.05	*p* > 0.05	*p* > 0.05	*p* > 0.05	*p* > 0.05	*p* > 0.05	*p* > 0.05	*p* > 0.05
	Bieliefs	*p* > 0.05	*p* > 0.05	*p* > 0.05	*p* > 0.05	*p* > 0.05	*p* > 0.05	*p* > 0.05	*p* > 0.05
	Empathy	*p* > 0.05	*p* > 0.05	*p* > 0.05	**r = 0.69** ***p* < 0.01**	*p* > 0.05	*p* > 0.05	*p* > 0.05	*p* > 0.05
	Comprehension	*p* > 0.05	*p* > 0.05	**r = 0.31** ***p* < 0.0**	*p* > 0.05	*p* > 0.05	*p* > 0.05	**η = 0.55** ***p* < 0.05**	**r = 0.24** ***p* < 0.05**
Emotion Comprehension Test	Situation	*p* > 0.05	*p* > 0.05	*p* > 0.05	*p* > 0.05	*p* > 0.05	*p* > 0.05	*p* > 0.05	**r = 0.21** ***p* < 0.05**
	Reaction	**r = 0.54** ***p* < 0.05**	*p* > 0.05	*p* > 0.05	*p* > 0.05	*p* > 0.05	*p* > 0.05	*p* > 0.05	**r = 0.31** ***p* < 0.05**
Hinting Task		*p* > 0.05	*p* > 0.05	**r = 0.61** ***p* < 0.05**	*p* > 0.05	**r = −0.33** ***p* < 0.05**	*p* > 0.05	**η = 0.42** ***p* < 0.05**	*p* > 0.05

Legend: significant results are in bold and have a gray background; AEDs—anti-epileptic drugs; *p*—level of significance; r—Pearson’s coefficient, η—coefficient used to assess the correlation between nominal and quantitative variables.

**Table 4:** Correlations between ToM test scores and demographic variables in the control group.

		Age	Sex	Years of Education	MoCA Scores
Faux Pas Test	Detection	*p* > 0.05	*p* > 0.05	*p* > 0.05	*p* > 0.05
	Understanding	*p* > 0.05	**η = 0.38** ***p* < 0.05**	*p* > 0.05	*p* > 0.05
	Intentions	*p* > 0.05	*p* > 0.05	*p* > 0.05	*p* > 0.05
	Bieliefs	*p* > 0.05	*p* > 0.05	*p* > 0.05	*p* > 0.05
	Empathy	*p* > 0.05	**η = 0.52** ***p* < 0.01**	*p* > 0.05	*p* > 0.05
	Comprehension	*p* > 0.05	*p* > 0.05	*p* > 0.05	*p* > 0.05
Emotion Comprehension Test	Situation	*p* > 0.05	**η = 0.51** ***p* < 0.05**	*p* > 0.05	*p* > 0.05
	Reaction	**r = 0.29** ***p* < 0.05**	**η = 0.43** ***p* < 0.05**	*p* > 0.05	*p* > 0.05
Hinting Task		*p* > 0.05	*p* > 0.05	*p* > 0.05	*p* > 0.05

Legend: significant results are in bold and have a gray background; *p*—level of significance; r—Pearson’s coefficient, η—coefficient used to assess the correlation between nominal and quantitative variables.

## Data Availability

The data presented in this study are available on reasonable request from the corresponding author.
